# Muscle strength trajectories and their association with postoperative health-related quality of life in patients undergoing coronary artery bypass grafting surgery: a prospective cohort study

**DOI:** 10.1186/s12872-023-03056-7

**Published:** 2023-01-16

**Authors:** Johanneke Hartog, Sandra Dijkstra, Willem Dieperink, Trynke Hoekstra, Joke Fleer, Lucas H. V. van der Woude, Pim van der Harst, Maarten Nijsten, Massimo A. Mariani, Fredrike Blokzijl

**Affiliations:** 1 Department of Cardiothoracic Surgery, University Medical Center Groningen (UMCG), University of Groningen, Hanzeplein 1, AB41, 9713 GZ Groningen, The Netherlands; 2 Department of Critical Care, University Medical Center Groningen, University of Groningen, Groningen, The Netherlands; 3 Research Group Nursing Diagnostics, Hanze University of Applied Sciences, Groningen, The Netherlands; 4 Department of Health Sciences and Amsterdam Public Health Research Institute, Vrije Universiteit Amsterdam, Amsterdam, The Netherlands; 5 Department of Health Psychology, University Medical Center Groningen, University of Groningen, Groningen, The Netherlands; 6 Department of Rehabilitation Medicine, Center for Human Movement Sciences, University Medical Center Groningen, University of Groningen, Groningen, The Netherlands; 7 Department of Cardiology, University Medical Center Groningen, University of Groningen, Groningen, The Netherlands; 8 Department of Cardiology, University Medical Center Utrecht, Utrecht, The Netherlands

**Keywords:** Cardiac surgical procedures, Health status, Quality of life, Sarcopenia, Muscular atrophy, Muscle strength, Muscle weakness, Therapeutics, Sex

## Abstract

**Background:**

Patients with sarcopenia have a higher risk of poor recovery after coronary artery bypass grafting (CABG). Little is known about the impact of changes in muscle strength (the primary indicator for sarcopenia) on health-related quality of life (HR-QoL). This study aimed to (1) identify subgroups with different muscle strength trajectories, (2) identify differences in preoperative risk factors among trajectory group membership, and (3) explore their prognostic value on postoperative HR-QoL in patients undergoing CABG.

**Methods:**

In this prospective observational study 131 patients undergoing elective CABG completed grip strength tests and HR-QoL questionnaires. Latent Class Growth Mixture Modelling (LCGMM) was used to identify clinically relevant trajectories (> 5% of study population) for weight-normalised grip strength, measured at admission, 3 days, and 6 months after surgery. Differences between trajectory group membership at baseline were evaluated. The impact of trajectory group membership on postoperative HR-QoL was evaluated with multiple linear regression models.

**Results:**

Due to low numbers (n = 15), female patients were excluded from LCGMM and subsequent statistical analyses. In males (n = 116), we identified two main weight-normalised grip strength trajectories: a “stable average” trajectory with a slight decline immediately post-surgery and recovery to preoperative levels (n = 85) and a “high” trajectory with a considerable immediate decline after surgery but followed towards a higher level of recovery compared to preoperative level (n = 27). The “stable average” patients were older (68 vs. 57 years; *P* = 0.003), had more diabetes (27% vs. 4%; *P* = 0.01) and had a higher BMI (27.8 vs. 24.8; *P* = 0.005) compared to the “high” group. After correction for age, diabetes, and baseline HR-QoL, group trajectory membership was not associated with postoperative HR-QoL, yet an increase in individual change scores of weight-normalised grip strength was associated with a better postoperative HR-QoL. We also identified one small trajectory group (n = 4, ≤ 5%).

**Conclusions:**

This study showed two relevant weight-normalised grip strength trajectories in male patients undergoing CABG, varying in important preoperative risk factors. While change scores of grip strength per weight did predict postoperative HR-QoL, the trajectory subgroups could not predict postoperative HR-QoL. Future research should focus on female patients, reacting potentially different on CABG and/or rehabilitation treatment.

*Trial registration* NCT03774342, 12-12-2018.

**Supplementary Information:**

The online version contains supplementary material available at 10.1186/s12872-023-03056-7.

## Background

Approximately 28% of patients undergoing coronary artery bypass grafting (CABG) have sarcopenia [1]. This multifactorial geriatric syndrome is considered a muscle disease with progressive and generalised loss of skeletal muscle strength as a principal determinant [2]. Additional components of sarcopenia are declines in muscle mass, muscle quality and physical performance [2]. Cardiac surgical patients with sarcopenia have longer hospital stays, a higher risk of major adverse cardiac events, and decreased long-term survival after cardiac surgery [3, 4]. Since sarcopenia is associated with ageing and more elderly patients are listed for major cardiac surgery, the role of sarcopenia will further increase in cardiac surgical treatment and rehabilitation care. This requires health-care professionals to search for optimal interventions to prevent adverse outcomes and improve patient-reported outcome measures, such as health-related quality of life (HR-QoL) [5].

As in many other research areas, HR-QoL is, nowadays, seen as an important outcome in cardiac surgery [6]. It reflects patients’ experience of the impact of the disease or treatment on their individual health on physical, social, and mental dimensions [7]. HR-QoL is, given the decrease in postoperative mortality, a useful complement to traditional clinical outcomes and is widely used, for example, when deciding whether or not to operate or when evaluating rehabilitation programmes [6, 8, 9]. Monitoring sarcopenia and understanding of the impact of sarcopenic parameters in CABG and how it affects post-operative HR-QoL, are needed to enhance the development of effective interventions in patients undergoing CABG [7].

The principal physical outcome of sarcopenia, muscle strength, is determined by muscle mass, quality, volume, length and activation level, and as such among others associated with aging and/or physical inactivity [1, 10]. (Weight-normalised) grip strength is a widely used indicator of muscle strength in elderly or clinical populations and can be easily and reliably determined with an inexpensive hand-held dynamometer [11, 12]. A person’s grip strength has prognostic value for all-cause death, cardiovascular disease, and postoperative complications after cardiac surgery [13, 14].

Despite a clear positive association between grip strength and HR-QoL is acknowledged in elderly [1], this association is not yet confirmed in CABG patients. Although, several studies have found associations between other physical outcomes, such as left ventricular function or unstable angina, and HR-QoL in CABG patients [6], a recent study found no association between preoperative or acute postoperative grip strength with immediate postoperative HR-QoL in this patient group [15]. This study, however, did not study associations between changes in preoperative and postoperative grip strength and HR-QoL. Interestingly, changes in preoperative and postoperative grip strength, or grip strength recovery, have been more predictive of postoperative complications after cardiac surgery than using only measures of preoperative grip strength [16]. Monitoring changes in grip strength over time thus provides a more important measure of surgical outcome, than just a single cross-sectional grip strength measurement.

Such changes in pre- and postoperative scores are often compared with each other on group level (mean ± SD), thus evaluating a single trajectory of the whole group. Since most groups consists of patients with various characteristics such as gender, preoperative risk factors, surgery parameters and so on, this approach may mask important diversity in recovery among patients. This heterogeneity of ‘increasers’ and ‘decreasers’ may be missed by evaluating only group means. In contrast, latent class growth mixture modelling (LCGMM) is a good alternative to determine subgroups within a given population with similar time courses or trajectories [17]. LCGMM may also provide more insight into how grip strength develops over time within potential subgroups, and how these are associated to postoperative recovery, functioning and HR-QoL. The present study aimed to (1) identify distinct trajectories of the development of grip strength over time (i.e., preoperative until 6 months after CABG); (2) identify differences in preoperative risk factors and preoperative sarcopenia parameters between trajectory groups; and (3) explore the prognostic value of these distinct subgroups on postoperative HR-QoL.

## Methods

### Design overview

This prospective single-centre cohort study was conducted in the University Medical Center Groningen (UMCG). Patients were identified and informed by the attending doctor or nurse practitioner on the date of admission, usually the day before surgery. After informed consent, patients were included and preoperative measurements were obtained. Postoperative assessment of muscle strength was performed in the hospital three days after surgery (short-term) and 6 months after surgery (long-term) at the patients’ homes. On these time points we also performed bioelectrical impedance analysis (BIA) as a potential indicator of muscle mass and quality. HR-QoL was measured at baseline and 6 months after surgery. All methods were performed in accordance with the Guidelines for Reporting on Latent Trajectory Studies (GRoLTS-checklist) and the STROBE-guidelines [18].

### Eligibility criteria

Adult patients admitted for elective, on-pump CABG in the University Medical Center Groningen, the Netherlands were considered for the study. Exclusion criteria were previous cardiac surgery and combined surgery, pre-existing neurological condition (i.e., dementia, stroke, epilepsy), psychiatric illness, pre-existing muscular diseases, or missing extremities (not possible to measure muscle mass of the extremities) and presence of an ICD or hip replacements because of interference with BIA equipment. When patients were likely to have difficulty understanding the Dutch language, they were also excluded from the study.

### Outcome measures

Grip strength as measure of muscle strength was tested with a Jamar Hydraulic hand-held dynamometer (Model 5030J1), which has good to excellent (r > 0.80) test–retest reliability [12]. To become familiar with the grip strength test, patients were asked to perform one practice-trial followed by three consecutive tests for each hand. The highest score of patients’ dominant hand of the handgrip test, which was normalized for preoperative weight, was used for analyses. Reference values were used to interpret the magnitude of grip strength [19].

HR-QoL was measured using the valid and reliable RAND-36 version-2 questionnaire [20]. This questionnaire is a widely used and validated instrument containing eight health domains: physical functioning (PF, ten items), social functioning (SF, two items), role limitations due to physical health problems (RP, four items), role limitations due to emotional problems (RE, three items), mental health (MH, five items), vitality (V, two items), bodily pain (BP, two items), and general health perception (GH, five items). Each domain is transformed to a scale between 0 and 100, a higher score is equals better health. Two summarized scores were calculated: a physical component score (PCS, which included PF, RP, BP, and GH) and a mental component score (MCS, which included MH, RE, SF, and V) [20, 21].

### Preoperative risk factors and preoperative sarcopenic parameters

Preoperative risk factors, including age, sex, body mass index, European System for Cardiac Operative Risk Evaluation II (EuroSCORE II), and the presence of comorbidities such as diabetes, pulmonary disease, arterial vascular disease, renal disease, and impaired ventricular function, were retrieved from the electronic patient medical records [21–23]. Definitions of these risk factors are included in Additional file 1: Table S1.

In addition to muscle strength, preoperative sarcopenic parameters included the secondary parameters of sarcopenia: muscle quantity and quality. Bioelectrical Impedance analysis at 50 kHz (BIA 101 Anniversary edition, AKERN, Florence, Italy) was used to determine the so-called appendicular skeletal muscle mass (ASMM) as an estimate of muscle quantity and BIA-derived phase angle (PA) as an estimate of muscle quality. Electrodes were placed on the hand and foot at the side while lying supine. The equation used are supplied in Additional file 1.

### Perioperative and postoperative characteristics

Perioperative data included duration of surgery, time on cardiopulmonary bypass (CPB), cross clamp time, and the number of (arterial) grafts. The following postoperative complications were collected: delirium, atrial fibrillation, myocardial infarction, surgical re-exploration, deep sternal wound infection, and renal failure all within 30 days after surgery and stroke/transient ischemic attack within 72 h after surgery [9, 21, 24–26]. Additional postoperative variables were duration of stay at the Intensive Care Unit and discharge destination. The definitions are included in the Additional file 1: Table S2.

### Statistical analyses

Descriptive statistics were used to present pre-, peri-, and postoperative characteristics. To identify subgroups of distinct trajectories of grip strength development over time LCGMM was performed using the ‘lcmm’ package in R (V. 4.1.2) [27]. The default mode of the argument ‘idiag’ of the ‘lcmm’ package was used, indicating unstructured variance–covariance matrix for the random effects [27]. An exploratory approach was chosen, meaning that as many classes as possible that yielded clinical relevant solutions were estimated [28]. First, quadratic trajectories were tested, which was expected to be the best representing pattern to the data [28]. Then, linear trajectories were tested for further exploration. Also, Latent Class Growth Modelling (LCGM, i.e., no allowance of within-class variances) were evaluated, however these models showed poorer fit with the data compared to LCGMM models (data not shown). Therefore, LCGMM models were chosen in favour of LCGM models. Time was coded ‘0 for preoperative’, ‘1 for three days’, and ‘2 for 6 months’. The ‘grid search’ function was used, in which the number of random start values was set on 100 with 30 final iterations. The model was selected based on model fit indices and clinical interpretability. The model fit indices included (1) a lower Bayesian Information Criteria (BIC), in which a difference of 10 points was considered as sufficient improvement [29], and (2) a higher average posterior probability of trajectory group membership (i.e. the probability to belong to a class), which should be greater than or equal to 0.7 for each subgroup [17]. Models with clinically interpretable solutions and larger groups were selected in favour of uninterpretable solutions and smaller groups [30]. Small trajectory-groups (≤ 5%) were not included in subsequent statistical analyses.

After model selection, differences in preoperative risk factors, sarcopenia parameters, and HR-QoL were first analysed between the identified subgroups using Pearson’s chi-squared test (binary or categorical variables) or (multiple or repeated) ANOVA using post-hoc tests with Bonferroni correction (continuous variables). Degrees of freedom were adjusted according to Greenhouse–Geisser when sphericity was violated. For binary or categorical variables, the Fisher’s exact test was used, when > 20% of the cells had an expected count less than 5. For continuous variables, the Kruskall-Wallis test with Dunn’s multiple-comparison post-hoc test (using a Bonferroni correction) was used when normality could not be assumed.

Second*,* the impact of trajectory group membership on postoperative HR-QoL at 6 months was evaluated by three (multiple) linear regression models*.* In the first model, univariable analyses were conducted in which trajectory group membership of grip strength (independent) was related to postoperative HR-QoL (dependent). The association was adjusted for age in model 2 and adjusted for the risk factors diabetes and baseline HR-QoL in model 3.

Additional analyses were performed to determine whether more ‘traditional’ statistical approaches of change scores would provide a stronger or weaker predication of postoperative HR-QoL by grip strength per weight. Individual change scores for weight-normalised grip strength were calculated as the value after 6 months divided by the preoperative value. Subsequently, the same regression analyses were performed with the change score as independent variable.

All analyses were performed separately for males and females, because of the different relationship between sarcopenic parameters and HR-QoL by sex [6, 31]. All analyses were tested 2-sided and *p*-values of < 0.05 were considered statistically significant. All data were analysed using Stata SE/17.0 (StataCorp LLC, revision April 2021, Lakeway, TX, USA) and SPSS version 23.0 (IBM Corp. Released 2015. IBM SPSS Statistics for Windows, Armonk NY).

## Results

**Fig. 1:**
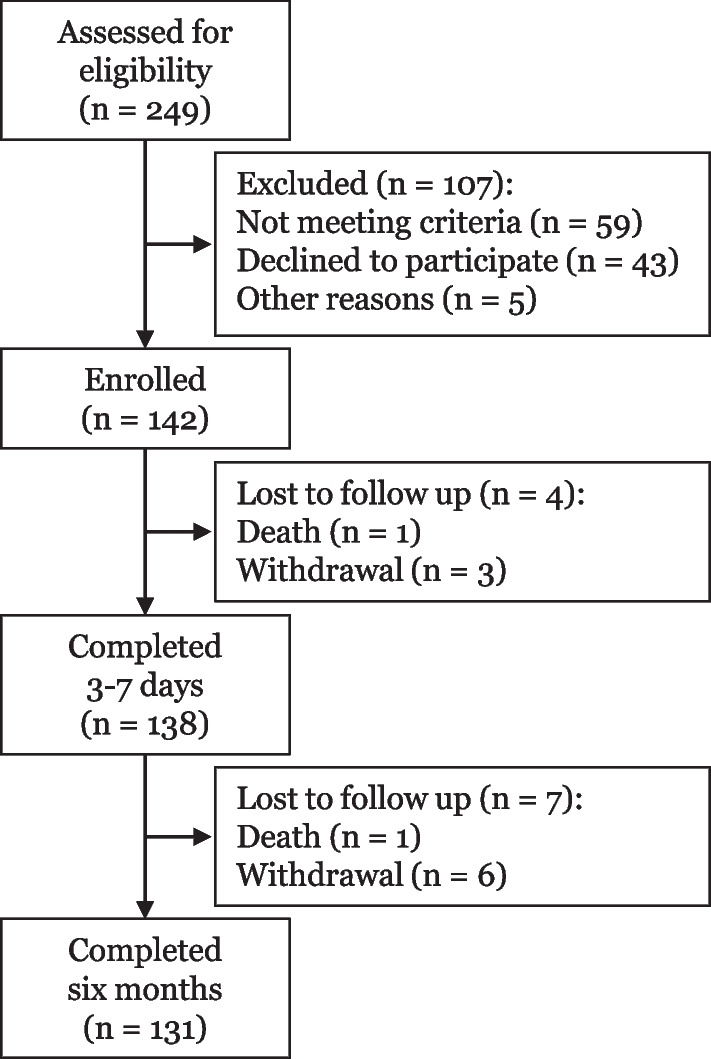
Flow chart of the present study

A total of 142 patients undergoing elective CABG enrolled in this prospective study between October 2018 and July 2019 (Fig. 1). Eleven patients were excluded from analysis, because two patients died and nine patients were lost to follow up (Additional file 1: Table S3). Table 1 and 2 present the baseline, peri-operative, and postoperative characteristics. One male did not dare to perform the grip strength test on day three after surgery. In addition, due to logistical reasons, this measurement could not be performed on one female, as she was transferred to another hospital as part of routinely standard care before measurements could be performed. Another three patients did not perform the grip strength test with the dominant hand at baseline, therefore the measurements of the non-dominant hand of these patients were used. In 33 patients (25%), the radial artery of the non-dominant hand was used for grafting. As a result, only one male performed the grip strength test with the hand whose arterial graft had been used. This patient did not show outlying grip strength values. Unfortunately, we were unable to obtain useful subsequent BIA measurements due to technical and methodological reasons. Direct post-operative measurements showed very high variation and were not reproducible and device malfunction made a number of measurements at 6 months. Due to low numbers of female patients (n = 15), LCGMM and the subsequent statistics (between-groups differences and linear regression) could not be performed in females. Additional file 2: Fig. S4 shows the individual changes of grip strength per weight and HR-QoL for females.

**Table 1:** Baseline characteristics of patients undergoing coronary artery bypass graft

Baseline characteristics	Female n = 15	Male n = 116	Total group n = 131
Age (years)	65 (58, 68)	67 (56, 72)	66 (56, 72)
BMI (kg/m^2^)	30.5 (28.0, 35.1)*	26.8 (24.5, 30.0)	27.1 (24.8, 30.8)
EuroSCORE II,	1.7 (1.3, 2.4)	1.5 (1.1, 2.2)	1.5 (1.1, 2.2)
Diabetes mellitus	4 (27%)	26 (22%)	30 (23%)
Pulmonary disease	3 (20%)	11 (10%)	14 (11%)
Arterial vascular disease	0 (0%)	7 (6%)	7 (5%)
Renal disease	1 (7%)	12 (10%)	13 (10%)
LVEF			
> 50%	0 (0%)	1 (1%)	1 (1%)
30–50%	4 (27%)	35 (30%)	39 (30%)
< 30%	11 (73%)	80 (69%)	91 (70%)
Preoperative sarcopenia parameters			
Grip strength (N/kg)	2.4 ± 0.6*	4.9 ± 1.1	4.6 ± 1.3
ASMM (kg)	22.2 (20.2, 22.8)*	26.0 (23.8, 28.2)^1^	25.6 (22.9, 28.0)
ASMM (kg/m^2^)	7.5 (7.2, 7.9)*	8.1 (7.4, 8.7)^1^	7.9 (7.4, 8.6)
Phase angle	6.2 ± 1.1	6.6 ± 0.9^1^	6.6 ± 0.9
Resistance	470.3 ± 35.0*	430.2 ± 63.5^1^	434.9 ± 62.1
Reactance	50.7 ± 9.5	49.6 ± 7.5^1^	49.7 ± 7.7
Preoperative Health-related quality of life			
PCS baseline^2,3^	51.1 (37.9, 61.8)*	65.6 (52.8, 81.2)	63.7 (50.1, 78.1)
MCS baseline^2,4^	61.9 (51.6, 71.8)*	76.8 (61.3, 90.1)	73.8 (60.1, 87.8)
PCS 6 months	68.1 (59.4, 92.5)	75.0 (60.9, 87.5)	74.9 (60.3, 88.4)
MCS 6 months	73.4 (50.2, 88.1)	80.3 (64.6, 88.9)	79.4 (64.5, 88.4)

Values are presented as n (% yes), mean ± SD or median (IQR)

*ASMM* appendicular skeletal muscle mass, *BMI* body mass index, *IQR* interquartile range, *LVEF* left ventricular ejection fraction, *MCS* mental component score, *PCS* physical component score

^1^Value for three patients unknown

^2^Score range: 0–100; a higher score is equivalent to better health-related quality of life

^3^Physical component score for two patients unknown. ^4^Mental component score for one patient unknown

*Significantly different compared to male patients (*P* < 0.05)

**Table 2:** Peri-operative and postoperative characteristics of patients undergoing coronary artery bypass graft

Peri-operative characteristics	Female n = 15	Male n = 116	Total group n = 131
Number of grafts			
One graft	0 (0%)	1 (1%)	1 (1%)
Two grafts	15 (100%)	113 (97%)	128 (98%)
Three grafts	0 (0%)	2 (2%)	2 (2%)
Number of arterial grafts			
No arterial graft	0 (0%)	3 (3%)	3 (2%)
Use of 1 arterial graft	11 (73%)	81 (70%)	92 (70%)
Use of 2 or more arterial grafts	4 (27%)	32 (27%)	36 (28%)
Surgical time (min)	244 (215, 279)	255 (229, 279)	253 (227, 279)
CPB-time^1^ (min)	107 (90, 121)	104 (85, 125)	106 (86, 123)
Cross clamp time^2^ (min)	60 (52, 78)	61 (47, 77)	61 (48, 78)
Postoperative characteristics			
Delirium	1 (7%)	5 (4%)	6 (4.6%)
Atrial fibrillation	0 (0%)	14 (12%)	14 (10.7%)
Myocardial infarction	0 (0%)	1 (19%)	1 (0.8%)
Surgical re-exploration	0 (0%)	2 (2%)	2 (1.5%)
Deep sternal wound infection	0 (0%)	2 (2%)	2 (1.5%)
Stroke/TIA	0 (0)	0 (0)	0 (0)
Renal failure	0 (0)	0 (0)	0 (0)
Prolonged ICU stay	4 (27%)	30 (26%)	34 (26%)^3^
Discharge destination			
Home	6 (40%)*	88 (76%)	94 (71.8%)
Other hospital	8 (53%)	10 (9%)	18 (13.7%)
Rehabilitation Centre	1 (7%)	18 (16%)	19 (14.5%)

Values are presented as n (% yes) or median (IQR)

*CPB* cardiopulmonary bypass, *ICU* intensive care unit, *IQR* interquartile range, *TIA* transient ischemic attack

^1^CPB-time for one patient unknown

^2^n = 124, 7 patients no use of CPB, but surgery was performed on beating heart

^3^Median (IQR): 21 (18, 24)

*Significantly different compared to male patients (*P* < 0.001)

### Male grip strength per weight trajectories

For males, the model fit indices for quadratic and linear LCGMM models with one to five trajectories are presented in Additional file 2: Table S4. Figures of the distinct trajectories of these models are shown in Additional file 2: Figs. S1 and S2. As expected, the models with quadratic trajectories showed better statistical fit compared to models with linear trajectories.

**Fig. 2:**
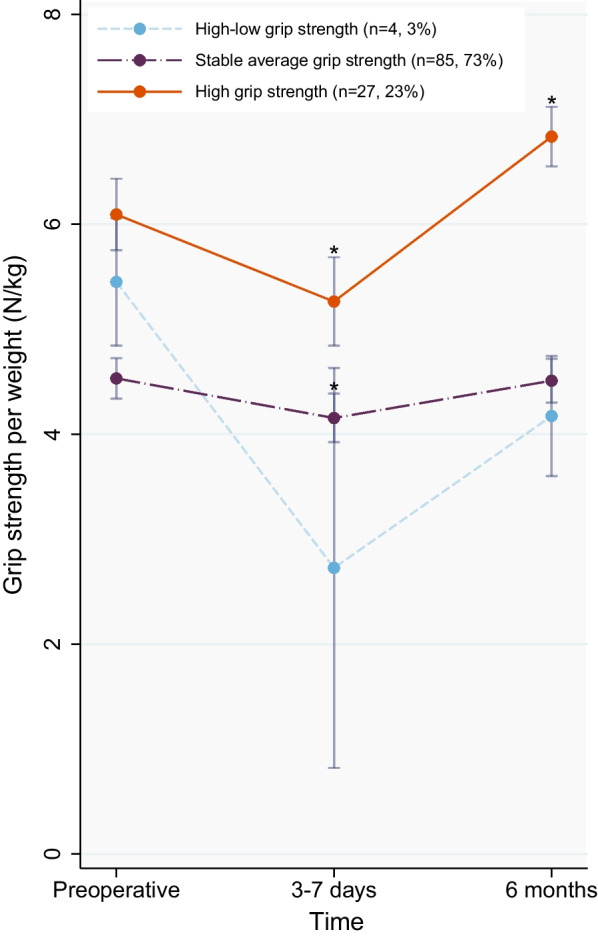
Three identified (weight-normalised) grip strength trajectories in men undergoing coronary artery bypass graft. Mean ± 95% Confidence Interval are presented; * Significantly different compared to preoperative (*P*-value < 0.001) Preoperative: 2 (IQR: 1–3) days before surgery; 3–7 days: 3 (IQR:3–3) days after surgery; 6 months: 193 (IQR:188–201) days after surgery. IQR: interquartile range

Based on the BIC, a two-class quadratic solution could be considered as best fit model, but class 1 consisted only of 3%, which was too small to be considered as clinically relevant (Additional file 2: Table S4, Figs. S1 and S2). Therefore, a three-class model was selected, which identified the two main trajectories: “stable average” grip strength (n = 85, 73%), “high” grip strength (n = 27, 23%), and one small trajectory: “high-low” grip strength (n = 4, 3%). The three trajectory groups are visualized in Fig. 2A and Additional file 2: Fig. S3. The small “high-low” group was not statistically explored (≤ 5%). In the “stable average” group, grip strength per weight showed a slight but significant decrease (from 4.5 to 4.2 N/kg, *P*-value < 0.001, F(1.8,153.0) = 23.3, Fig. 2) and increased to the same preoperative level at 6 months after surgery (from 4.5 to 4.5 N/kg, *P*-value = 1.000, F(1.8,153.0) = 23.3). The “high” grip strength group had the highest preoperative values but showed a considerable and significant decrease of 13% after surgery (from 6.1 to 5.3 N/kg, *P*-value < 0.001, F(1.6,19.0) = 98.2). Subsequently, the values rose to a significantly higher level compared to preoperatively (from 6.1 to 6.8 N/kg, *P*-value < 0.001, F(1.6,19.0) = 98.2). Table 3 shows the preoperative risk factors and preoperative sarcopenia parameters for the 2 main trajectories. The “stable average” patients were significantly older (68 vs. 57 years; *P* = 0.003), had more diabetes (27% vs. 4%; *P* = 0.01) and had a higher BMI (27.8 vs. 24.8; *P* = 0.005) compared to the “high” grip strength group. In contrast, the left ventricular ejection fraction was higher in the “stable average” group compared to the “high” grip strength group. Also, ASMM seemed higher in the “stable average” group compared to the “high” grip strength group, but this difference was not significant (8.2 vs. 7.7; *P* = 0.069). The preoperative sarcopenic parameters, grip strength, phase angle, and reactance were significantly lower in the “stable average” group compared to the “high” grip strength group (*P* < 0.05, Table 3).

**Table 3:** Differences in preoperative risk factors and preoperative sarcopenia parameters among trajectory group membership

	High-low grip strength	Stable average grip strength	High grip strength	*P*-value^1^
	n = 4 (3%)	n = 85 (73%)	n = 27 (23%)	
*Preoperative risk factors*				
Age (years)	63.0 (57.5, 71.5)	68.0 (58.0, 74.0)	57.0 (51.0, 68.0)	0.003^2^
BMI (kg/m^2^)	27.5 (26.3, 29.1)	27.8 (25.6, 30.9)	24.8 (21.7, 26.1)	0.005^2^
Diabetes mellitus	2 (50%)	23 (27%)	1 (4%)	0.010^3^
Pulmonary disease	0 (0%)	9 (11%)	2 (7%)	1.000^4^
Arterial vascular disease	0 (0%)	7 (8%)	0 (0%)	0.190^4^
Renal disease	0 (0%)	11 (13%)	1 (4%)	0.290^4^
LVEF = < 50%	0 (0%)	22 (26%)	14 (52%)	0.012^3^
EuroSCORE II	1.7 (1.4, 2.3)	1.5 (1.1, 2.3)	1.3 (1.1, 1.9)	0.093^2^
*Preoperative sarcopenia parameters*				
Grip strength (N/kg)	5.5 ± 0.4	4.5 ± 0.9	6.1 ± 0.9	< 0.001^5^
ASMM (kg)	26.0 (23.0, 27.1)	26.1 (24.4, 28.8)	24.7 (22.9, 27.4)	0.110^2^
ASMM (kg/m^2^)	7.8 (7.2, 8.8)	8.2 (7.6, 8.8)	7.7 (7.3, 8.4)	0.069^2^
Phase angle (PA)	6.8 ± 0.7	6.5 ± 0.9	7.0 ± 0.9	0.030^5^
Resistance (Rz)	456.1 ± 77.3	427.7 ± 65.7	434.4 ± 54.8	0.640^5^
Reactance (Xc)	54.6 ± 8.2	48.4 ± 7.1	52.7 ± 7.6	0.010^5^
*Preoperative Health-related quality of life*				
PCS baseline^6^	62.6 (45.6, 75.6)	63.7 (53.8, 78.5)	70.3 (57.5, 83.4)	0.280^2^
MCS baseline^6^	78.6 (46.4, 95.5)	76.9 (62.3, 90.1)	73.3 (59.8, 83.4)	0.510^2^
PCS 6 months^6^	57.2 (50.6, 74.7)	73.6 (60.6, 88.6)	77.2 (65.9, 86.9)	0.630^2^
MCS 6 months^6^	63.6 (35.5, 82.5)	80.9 (69.2, 89.7)	75.8 (57.8, 85.6)	0.200^2^

Values are presented as n (% yes), median (IQR), or mean ± SD

*ASMM* appendicular skeletal muscle mass, *BMI* body mass index, *IQR* interquartile range, *LVEF* left ventricular ejection fraction, *MCS* mental component score, *PCS* physical component score

^1^Difference between “stable average” and “high” grip strength trajectory

^2^Wilcoxon rank-sum

^3^Pearson’s chi squared test

^4^Fisher’s exact test

^5^Two sample t test

^6^Score range: 0–100; a higher score is equivalent to better health-related quality of life

### Impact of trajectory group membership on postoperative HR-QoL

The group comparison in Table 3 showed no statistical differences for postoperative HR-QoL. Results of the (multiple) regression analysis of the association between trajectory group membership and postoperative HR-QoL (Table 4) stressed the absence of an association between trajectory group membership and HR-QoL, also after (significant) correction for age, diabetes, and baseline HR-QoL.

**Table 4:** Linear regression parameters for associations of ‘grip strength per weight’-trajectory membership with postoperative health-related quality of life after coronary artery bypass grafting

	Grip strength per weight (N/kg)	Trajectory		
		Physical component score		
		Beta	95% CI	*P*-value
Model 1: Univariable	Stable vs high grip strength	− 0.065	− 0.254 to 0.124	0.496
Model 2: Age	Stable vs high grip strength	− 0.058	− 0.256 to 0.139	0.584
	Age	− 0.025	− 0.222 to 0.172	0.803
Model 3: Age, HR-QoL, and DB	Stable vs high grip strength	0.074	− 0.099 to 0.248	0.398
	Age	− 0.163	− 0.328 to 0.008	0.061
	Diabetes	− 0.340	− 1.216 to − 0.410	< 0.001
	Baseline PCS or MCS	0.428	0.261 to 0.585	< 0.001

		Mental component score		
		Beta	95% CI	*P*-value
Model 1: Univariable	Stable vs high grip strength	0.099	− 0.089 to 0.287	0.300
Model 2: Age	Stable vs high grip strength	0.054	− 0.140 to 0.248	0.583
	Age	0.161	− 0.033 to 0.355	0.103
Model 3: Age, HR-QoL, and DB	Stable vs high grip strength	0.099	− 0.059 to 0.256	0.218
	Age	− 0.009	− 0.164 to 0.146	0.912
	Diabetes	− 0.259	− 0.405 to − 0.103	0.001
	Baseline PCS or MCS	0.569	0.412 to 0.708	< 0.001

*CI* Confidence Interval, *HR-QoL* Health-Related Quality of Life, *DB* Diabetes, *MCS* mental component score, *PCS* physical component score

### Additional analyses

In contrast to trajectory membership, the individual relative change score for weight-normalized grip strength (i.e., values of 6 months divided by baseline) was positively associated with the PCS of postoperative HR-QoL but was not associated with the MCS of postoperative HR-QoL (Table 5). These associations were also shown after correction for age, diabetes, and baseline scores of the PCS and MCS.

**Table 5:** Linear regression parameters for associations of ‘grip strength per weight’-change scores (N/kg) with postoperative health-related quality of life after coronary artery bypass grafting

	Grip strength per weight (N/kg)	Physical component score		
		Beta	95% CI	*P*-value
Model 1: Univariable	Relative change scores^1^	0.263	0.079 to 0.444	0.005
Model 2: Age	Relative change scores^1^	0.262	0.076 to 0.446	0.010
	Age	− 0.008	− 0.192 to 0.177	0.935
Model 3: Age, HR-QoL, and DB	Relative change scores^1^	0.216	0.058 to 0.369	0.007
	Age	− 0.107	− 0.260 to 0.051	0.185
	Diabetes	− 0.298	− 1.090 to − 0.334	< 0.001
	Baseline PCS or MCS	0.408	0.246 to 0.559	< 0.001

		Mental component score		
		Beta	95% CI	*P*-value
Model 1: Univariable	Relative change scores^1^	0.040	− 0.149 to 0.229	0.677
Model 2: Age	Relative change scores^1^	0.063	− 0.125 to 0.251	0.509
	Age	0.184	− 0.004 to 0.372	0.055
Model 3: Age, HR-QoL, and DB	Relative change scores^1^	0.047	− 0.101 to 0.192	0.535
	Age	0.029	− 0.122 to 0.178	0.711
	Diabetes	− 0.226	− 0.368 to − 0.075	0.003
	Baseline PCS or MCS	0.571	0.413 to 0.710	< 0.001

*CI* Confidence Interval, *HR-QoL* Health-Related Quality of Life, *DB* Diabetes

^1^Value 6 months divided by preoperative value

## Discussion

The aim of this study was to identify different trajectories of weight-normalised muscle strength in patients undergoing CABG. Subsequently, the characteristics of these trajectories and their prognostic value on postoperative HR-QoL were examined, although this was not possible in the small group of females (n = 15). In males (n = 116), we identified two main trajectories. In 85 patients (73%) we observed a “stable average” pathway with a slight decrease followed by recovery to preoperative levels. In 27 patients (23%) we saw a “high” trajectory with a significant decrease of 13% immediately after surgery but a stronger recovery compared to preoperative levels. Preoperative risk factors (i.e., sarcopenic parameters, age, diabetes, and obesity), were more prevalent in the “stable average” trajectory group than in the “high” grip strength group. Trajectory group membership was, however, not a significant predictor of postoperative HR-QoL, nor for physical or mental component scores. Individual change scores in weight-normalized grip strength were however significantly associated with HR-QoL, also after correction for age, diabetes, and baseline HR-QoL.

Two main and a small subgroup were defined with distinct quadratic time courses. The slight decrease in grip strength in largest trajectory group (73%) is comparable with the observations by Fu and colleagues [16] in which grip strength of cardiac surgical patients was almost fully recovered at the third postoperative day. Similarly, Teng and colleagues [3] showed a stable trajectory of grip strength up to one year after cardiac surgery. In contrast, Da Silva and colleagues [14] showed a significantly reduced grip strength at hospital discharge in cardiac surgical patients. Such decline was also seen in the second subgroup; the “high” grip strength group (23%). This group had preoperatively a higher score compared to healthy age-matched references [19], suggesting a positive patient selection. A relatively large decline in muscle strength was seen in this group. However, this group showed a high capacity to recover as the values at 6 months were higher compared to the preoperative values. The small “high-low” group (n = 4) seemed—despite having a high preoperative grip strength—to follow a less favourable trajectory, as the grip strength decreased severely immediately after surgery, and it seemed that they did not reach preoperative level. Although all four patients experienced at least one postoperative complication, we could not find one unique reason for being in this subgroup. Moreover, the sample size of this group was too small to elaborate further analyses.

To our knowledge, this is the first study to investigate the effects of distinct trajectories of muscle strength on postoperative HR-QoL in patients undergoing CABG. LCGMM has proven to be an adequate and advanced statistical technique to identify meaningful groups or classes of individuals over time [32]. This can be meaningful when tailoring the treatment. Also in the present study, the main trajectory subgroups differed on important preoperative risk factors (e.g., sarcopenic parameters, age, diabetes and obesity), known to affect surgical outcomes and postoperative HR-QoL [6, 8, 15]. Unexpectedly, grip strength trajectory group membership and postoperative HR-QoL were not associated in our results. Despite significant group differences in grip strength at each time point, the trajectories are possibly less distinctive. First, the BIC-values of the models for 1 trajectory and 3 trajectories differed by less than 10 points (which was considered as sufficient improvement). Second, LCGMM allows some variation within subgroups, which is shown in Additional file 2: Fig. S3A, B. LCGMM was, however, chosen in favour of Latent Class Growth Modelling (LCGM, i.e., no allowance of within-class variances), due to higher BIC-values that indicate a better fit with the data. Secondly, a positive selection of patients was possibly included, which potentially impacted our results. Our study group showed higher levels of grip strength as well as HR-QoL at baseline and at 6 months compared to literature [6, 8, 19, 32]. In addition, there was a tendency of lower preoperative grip strength values and HR-QoL in dropouts compared to included patients (*P*-value range: 0.095–0.170 with low sample size, n = 11, Additional file 1: Table S3). A wider spread and lower values would increase the variance and could lead to more distinct trajectory groups. On the other hand, older age, high BMI, and male sex are typical characteristics for patients undergoing CABG, indicating a representative population in our study [4, 14–16].

The present study was limited by low numbers of females (n = 15), allowing no separate trajectory analyses. Although there is a clear role for sex in the measurement of muscle strength measures, the effects of CABG on muscle strength and HR-QoL in female patients have been understudied, as fewer female patients undergo CABG [19]. In agreement with previous studies [6, 31], our study indicates that females have lower muscle strength and HR-QoL (Table 1, Additional file 2: Fig. S4). With growing evidence that gender differences must be taken into account at all stages of cardio- and/or rehabilitation-therapeutic strategies [33], future research should stratify analyses by sex. In addition, multi-centre studies are needed to ensure enough female patients.

A limitation of this study was, that we were not able to present the presence of sarcopenia in more detail. Regrettably, BIA measurements produced implausible results post-surgery and were hampered by practical problems for a number of follow-up measurements. Furthermore, ASMM at baseline showed a non-significant but opposite relation with grip strength at baseline. Also, based on the revised European Working Groups on Sarcopenia in Older People (EWGSOP) 2018 guidelines [2], the prevalence of low muscle mass in our study was only 3% (contrary to 50% according to the original EWGSOP 2010 guidelines [34]). This small subgroup was not suitable for further analyses. Meaningful cut-off levels to identify sarcopenia remain to be established for this study population. While the original EWGSOP guidelines proposed low muscle mass as the primary parameter for diagnosing sarcopenia, which was determined using the BIA-based equations and cut-off values of Janssen et al. (2000 [35], 2004 [36]), the current EWGSOP advises muscle strength as the primary parameter, while muscle mass is determined by different equations and cut-off values (i.e., Sergi et al. [37] and Gould et al. [38], respectively). A recent systematic review confirmed lower levels of sarcopenia among older adults when using revised EWGSOP guidelines [39]. Moreover, sarcopenia according to the revised EWGSOP 2018 guidelines seemed to be worse at predicting adverse outcomes, such as risk of hospitalisation and mortality. Possibly, BIA may be less suitable for use in surgical populations with major fluid shifts and/or obesity, as both factors are known to considerably affect the parameters generated by this device [40].

An important clinical finding was that patients with high preoperative grip strength experience a considerable decline in grip strength a few days after surgery. Because changes in grip strength per weight are associated with HR-QoL and most improvements in HR-QoL are shown during the first 2 months after CABG [32], future research should examine the recovery of grip strength and its impact on HR-QoL at shorter time intervals and earlier than 6 months after surgery. Because the immediate decrease in grip strength after CABG may impact HR-QoL at an earlier moment, possible different treatment strategies i.e., preoperative or early rehabilitation may be suitable for subgroups of patients.

Besides grip strength, future research should focus on a broader context of rehabilitation outcomes. For example, evaluation of strength of the lower extremities could be of added value, as hospital immobility can affect the lower extremities more severely [41, 42].

## Conclusions

This prospective study showed two relevant weight-normalised grip strength trajectories in male patients undergoing CABG, varying in important preoperative risk factors. While change scores of grip strength per weight did predict postoperative HR-QoL, the trajectory subgroups could not predict postoperative HR-QoL. Changes in easy-to-measure hand grip muscle strength are thus clinically relevant for postoperative HR-QoL. Pre- and postoperative rehabilitation could further enhance level and stability of muscle strength over time, potentially improving surgical outcome and HR-QoL. Future research should focus on female patients, reacting potentially different on CABG and/or rehabilitation treatment and on the development of easy clinical ways to identify sarcopenia that are reliable for surgical populations.

## Supplementary Information

**Additional file 1.** Definitions and equations of preoperative risk factors and postoperative complications. Characteristics of patients who dropped out and who completed the study.

**Additional file 2.** Results of Latent Class Growth Mixture Models (LCGMM) for grip strength per weight in males and figures of individual trajectories before and after coronary artery bypass grafting for grip strength per weight and Health-related Quality of Life (HR-QoL) in females.

## Data Availability

The datasets used and/or analysed during the present study are available from the corresponding author on reasonable request.

## Abbreviations

ASMM: Appendicular skeletal muscle mass
BIA: Bioelectrical impedance analysis
BIC: Bayesian information criteria
BMI: Body mass index
CABG: Coronary artery bypass grafting
CPB: Cardiopulmonary bypass
EWGSOP: European Working Group on Sarcopenia in Older People
HR-QoL: Health-related quality of life
LCGMM: Latent class growth mixture modelling
MCS: Mental component score
PA: Phase angle
PCS: Physical component score
RI: Resistive index
Rz: Resistance
UMCG: University Medical Center Groningen
Xc: Reactance
