# Supplementary material for: Muscle strength trajectories and their association with postoperative health-related quality of life in patients undergoing coronary artery bypass grafting surgery: a prospective cohort study
Source: BMC Cardiovasc Disord. 2023 Jan 16;23:20. doi: 10.1186/s12872-023-03056-7 (PMC9841699; doi:10.1186/s12872-023-03056-7)
Supplement: Supplementary file 1 — Additional file 1. Definitions and equations of preoperative risk factors and postoperative complications. Characteristics of patients who dropped out and who completed the study. [file 12872_2023_3056_MOESM1_ESM.docx]

**Additional file 1**

Table S1: Definitions of preoperative risk factors

| **Preoperative risk factors** |
| --- |
| *Diabetes*  Oral therapy or insulin dependent diabetes |
| *Pulmonary disease*  Prolonged use of steroids or other lung medication |
| *Arterial vascular disease*  Peripheral or abdominal vascular pathology or operation due to arterial vascular disease |
| *Renal disease*  A reduced renal function prior to surgery with an estimated Glomerular Filtration rate (eGFR)  <60 ml/min/1.73 m^2^ |
| *Ventricular function*  Left ventricular ejection fraction good >50%, moderate 30-50% or poor <30% |

Table S2: Definitions of postoperative complications

| **Postoperative complications** |
| --- |
| *Delirium during hospital admittance*  1) A Delirium Observation Screening (DOS)-score ≥ 3 at hospital ward and/or 2) A positive  Confusion Assessment Method for the Intensive Care Method (CAM-ICU) score at the ICU  and/or 3) Diagnosis confirmed by a psychiatrist or geriatrist according to the DSM-IV criteria |
| *Atrial fibrillation*  New onset of atrial fibrillation or atrial flutter requiring medical treatment or cardioversion within 30 days after surgery |
| *Myocardial infarction (MI) in the postoperative period*  Myocardial infarction associated with CABG (within 48 hours after CABG) is arbitrarily defined by elevation of cardiac biomarker values >10 x 99th percentile upper reference limit (URL) in patients with normal baseline cardiac troponin values. In addition, either (i) new pathological Q waves or new LBBB, or (ii) angiographic documented new graft or new native coronary artery occlusion, or (iii) imaging evidence of new loss of viable myocardium or new regional wall motion abnormality. After 48 hours, the standard definition of myocardial infarction is appropriate. The following criteria meets the diagnosis for MI: detection of a rise and/or fall of cardiac biomarker values, preferably cardiac troponin, with at least one value above the 99th percentile URL and in addition, either (i) symptoms of ischaemia, or (ii) new or presumed new significant ST- segment–T wave (ST–T) changes or new left bundle branch block (LBBB), or (iii) development of pathological Q waves in the ECG, or (iiii) imaging evidence of new loss of viable myocardium or new regional wall motion abnormality or identification of an intracoronary thrombus by angiography or autopsy |
| *Surgical re-exploration*  Thoracotomy due to bleeding, cardiac tamponade or graft failure within 30 days after surgery |
| *Deep wound infection*  When within 30 days after surgery deeper tissues are affected (muscle, sternum and  mediastinum) and one or more of the following three criteria are met:  1) surgical drainage or refixation  2) an organism is isolated from culture of mediastina tissue or fluid  3) antibiotic treatment because of a sternal wound |
| *Stroke*  An acute neurological event within 72 hours after surgery with focal signs and symptoms and without evidence supporting any alternative explanation. Diagnoses of stroke requires confirmation by a neurologist |
| *Renal failure*  When within 30 days after surgery one or more of the following criteria are met:  1) renal replacement therapy (dialysis or CVVH) which was not present preoperatively  2) highest postoperative creatinine level > 177 μmol/L and a doubling of the  preoperative value (the preoperative creatinine value is the value on which the  EuroSCORE is calculated) |
| *Prolonged ICU stay*  An ICU stay longer than 24 hours |

**BIA-device based equations for appendicular skeletal muscle mass and phase angle**

The cross-validated equation 1(1) was used to calculate the preoperative appendicular skeletal muscle mass (ASMM,) which is based on a healthy European elderly population and recommended in the revised EWGSOP guidelines(2). In equation 1, the resistive index (RI, i.e. height in centimetres squared/Rz), was used to normalize for height. The phase angle was calculated using the raw BIA values Resistance (Rz) and Reactance (Xc) (Equation 2), when it could not be derived from the BIA.

$$Equation 1:$$

$$ASMM= -3.964 + (0.227*RI) + (0.095*weight) + (1.384*sex) + (0.064*Xc)$$

$$Equation 2:$$

$$PA (degrees)=\left( \frac{Rz}{Xc} \right)x (180/\pi)$$

References

1. Sergi G, De Rui M, Veronese N, Bolzetta F, Berton L, Carraro S, et al. Assessing appendicular skeletal muscle mass with bioelectrical impedance analysis in free-living Caucasian older adults. Clin Nutr [Internet]. 2015;34(4):667–73. Available from: http://dx.doi.org/10.1016/j.clnu.2014.07.010

2. Cruz-Jentoft AJ, Bahat G, Bauer J, Boirie Y, Bruyère O, Cederholm T, et al. Sarcopenia: Revised European consensus on definition and diagnosis. Age Ageing. 2019;48(1):16–31.

Table S3: Relevant variables between patients who dropped out (n=11) and patients who completed the study (n=131)

| Baseline characteristics | Study completed  n = 131 | Drop out / deceased  n = 11 | P-value |
| --- | --- | --- | --- |
| Age (years) | 66.0 (56.0, 72.0) | 61.0 (52.0, 71.0) | 0.450 |
| BMI (kg/m2) | 27.1 (24.8, 30.8) | 28.4 (23.9, 34.2) | 0.450 |
| EuroSCORE II, | 1.5 (1.1, 2.2) | 1.9 (1.1, 2.9) | 0.350 |
| Diabetes mellitus | 30 (23%) | 2 (18%) | 0.720 |
| Pulmonary disease | 14 (11%) | 1 (9%) | 0.870 |
| Arterial vascular disease | 7 (5%) | 0 (0%) | 0.430 |
| Renal disease | 13 (10%) | 1 (9%) | 0.930 |
| LVEF > 50%  30-50%  < 30% | 1 (1%)  39 (30%)  91 (70%) | 0 (0%)  6 (55%)  5 (46%) | 0.170 |
| Preoperative Health-related quality of life | |  |  |
| Physical Component Score^1,2^ | 63.7 (50.1, 78.1) | 58.4 (43.1, 63.1) | 0.120 |
| Mental Component Score^1,3^ | 73.8 (60.1, 87.8) | 63.4 (55.6, 78.1) | 0.095 |
| Preoperative sarcopenia parameters | | | |
| Grip strength (N/kg) | 41.2±11.4 | 36.3±12.4 | 0.170 |
| ASMM (kg/m2) | 7.9 (7.4, 8.6) | 7.7 (7.1, 9.2) | 0.860 |
| Phase Angle | 6.6±0.9 | 6.5±1.3 | 0.910 |
| Peri-operative characteristics | | | |
| Total grafts One graft  Two grafts  Three grafts | 1 (1%)  128 (98%)  2 (2%) | 0 (0%)  11 (100%)  0 (0%) | 1.000 |
| Surgical time (min) | 253.0 (227.0, 279.0) | 255.0 (226.0, 283.0) | 0.780 |
| Postoperative characteristics | | | |
| Delirium | 6 (5%) | 1 (9%) | 0.510 |
| Atrial fibrillation | 14 (11%) | 0 (0%) | 0.250 |
| Myocardial infarction | 1 (1%) | 1 (9%) | 0.024 |
| Surgical re-exploration | 2 (2%) | 1 (9%) | 0.094 |
| Deep sternal wound infection | 2 (2%) | 1 (9%) | 0.094 |
| Stroke/TIA | 0 (0%) | 0 (0%) | 1.000 |
| Renal failure | 0 (0%) | 0 (0%) | 1.000 |
| Prolonged ICU stay | 34 (26%) | 4 (36%) | 0.490 |
| Discharge destination  Home  Other hospital  Rehabilitation centre  Nursing home  Deceased | 94 (72%)  18 (14%)  19 (15%)  0 (0%)  0 (0%) | 5 (46%)  3 (27%)  0 (0%)  1 (9%)  2 (18%) | <0.001 |

Values are presented as n (% yes), mean±SD or median (IQR). ^1^ Score range: 0-100; a higher score is equivalent to better health-related quality of life; ^2^ Physical component score for four patients unknown; ^3^ Mental component score for three patient unknown; BMI: body mass index; CPB: cardiopulmonary bypass; ICU: intensive care unit; IQR: interquartile range; LVEF: left ventricular ejection fraction; TIA: transient ischemic attack.
