# Supplementary material for: Muscle strength trajectories and their association with postoperative health-related quality of life in patients undergoing coronary artery bypass grafting surgery: a prospective cohort study
Source: BMC Cardiovasc Disord. 2023 Jan 16;23:20. doi: 10.1186/s12872-023-03056-7 (PMC9841699; doi:10.1186/s12872-023-03056-7)
Supplement: Supplementary file 2 — Additional file 2. Results of Latent Class Growth Mixture Models (LCGMM) for grip strength per weight in males and figures of individual trajectories before and after coronary artery bypass grafting for grip strength per weight and Health-related Quality of Life (HR-QoL) in females. [file 12872_2023_3056_MOESM2_ESM.docx]

**Additional file 2**

Table S4: Model fit parameters of LCGMM models for grip strength per weight in Males (n=116)

| **Grip strength per weight (N/kg)** | | | | | | | | | | | | | |
| --- | --- | --- | --- | --- | --- | --- | --- | --- | --- | --- | --- | --- | --- |
| **Allowance of within-class variances for intercept and –slope (LCGMM)** | | | | | | | | | | | | | |
|  | **BIC** | **Entropy** | **Average posterior propability per class^2^** | | | | | **Group membership per class (%)^3^** | | | | |  |
| **Quadratic** | | | **1** | **2** | **3** | **4** | **5** | **1** | **2** | **3** | **4** | **5** |  |
| 1 | 862.9 | 1.0 | 1 |  |  |  |  | 100 |  |  |  |  |  |
| 2 | 857.2 | 1.0 | 0.970 | 0.997 |  |  |  | 3 | 97 |  |  |  |  |
| **3** | **869.9** | **0.8** | **0.849** | **0.977** | **0.923** |  |  | **23** | **3** | **73** |  |  |  |
| 4 | 884.3 | 0.8 | 0.919 | 0.985 | 0.797 | 0.788 |  | 76 | 3 | 16 | 5 |  |  |
| 5 | 894.9 | 0.8 | 0.878 | 0.908 | 0.910 | 1 | 0.882 | 23 | 24 | 48 | 1 | 3 |  |
| **Linear** | | | **1** | **2** | **3** | **4** | **5** | **1** | **2** | **3** | **4** | **5** |  |
| 1 | 950.6 | 1.0 | 1 |  |  |  |  | 100 |  |  |  |  |  |
| 2 | 963.6 | 0.5 | 0.768 | 0.869 |  |  |  | 28 | 72 |  |  |  |  |
| 3 | 972.3 | 0.8 | 0.973 | 0.909 | 0.879 |  |  | 2 | 71 | 28 |  |  |  |
| 4 | 983.6 | 0.7 | 0.998 | 0.781 | 0.893 | 0.907 |  | 2 | 45 | 36 | 17 |  |  |
| 5 | 992.3 | 0.8 | 0.922 | 0.861 | 0.909 | 1 | 0.875 | 28 | 41 | 8 | 2 | 21 |  |

LCGMM: Latent class growth mixture modelling


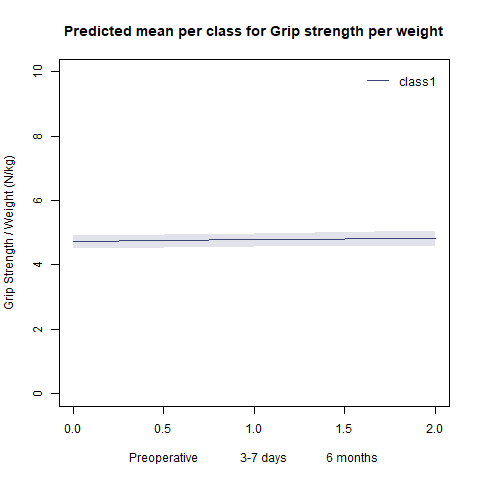

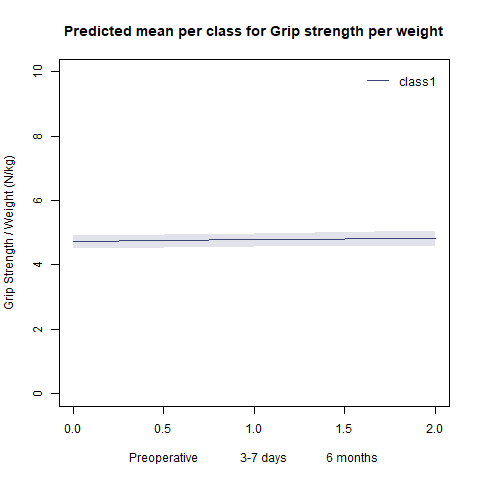

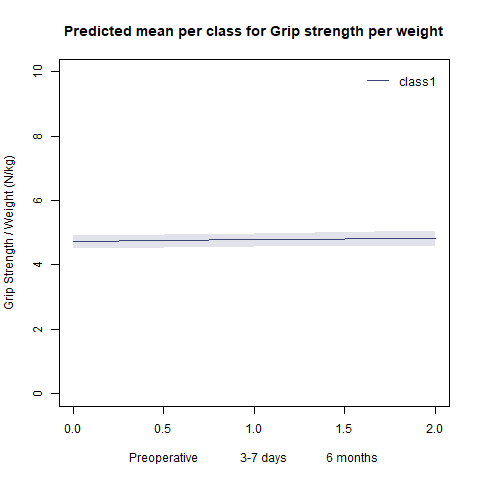

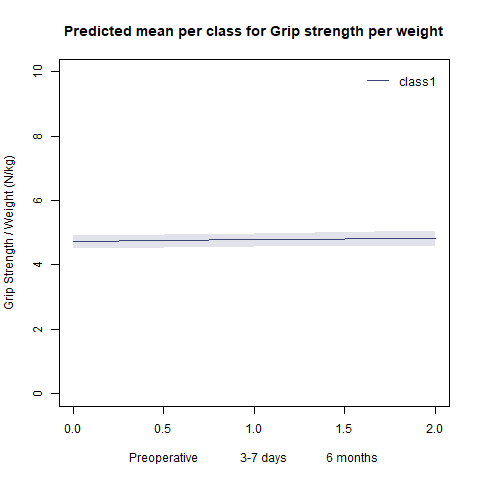

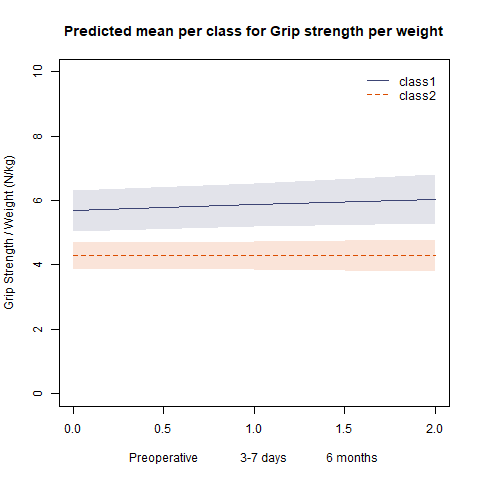

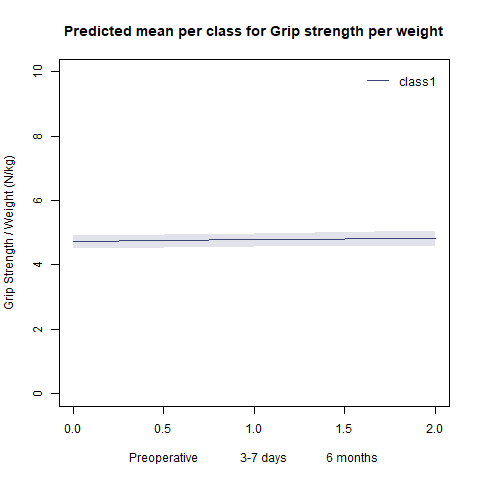

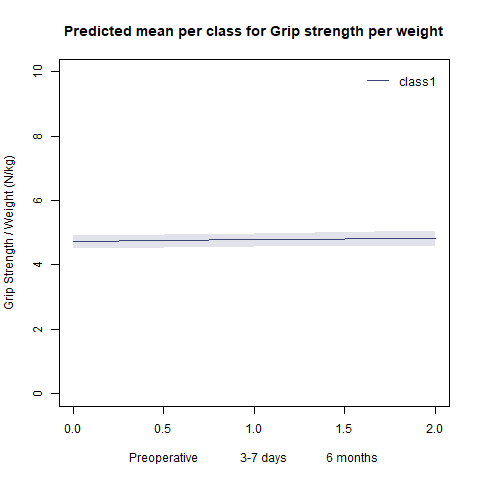

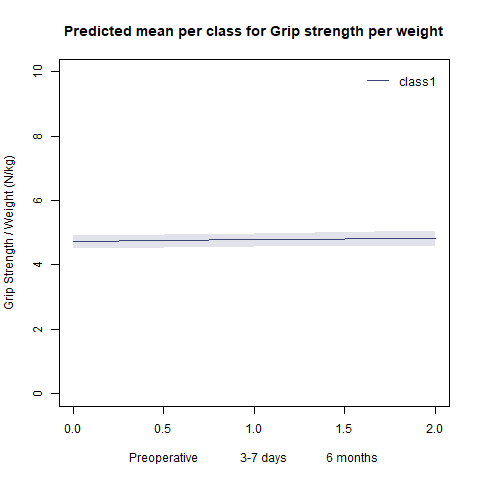

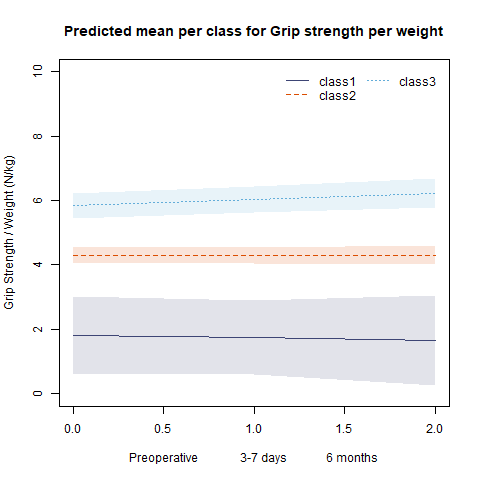

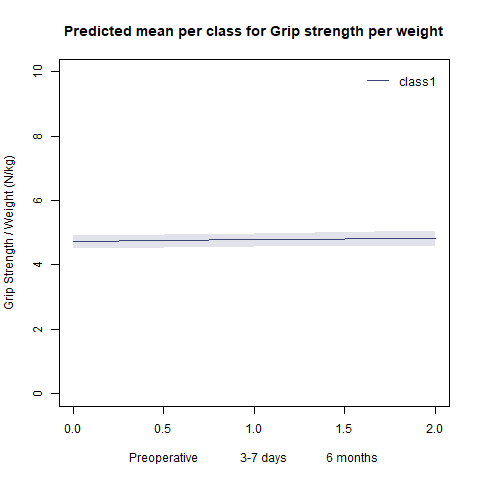

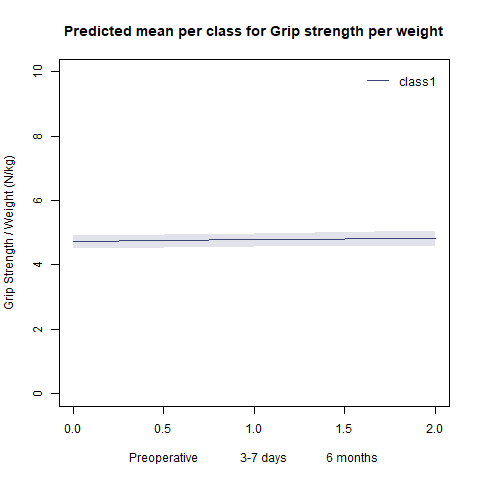

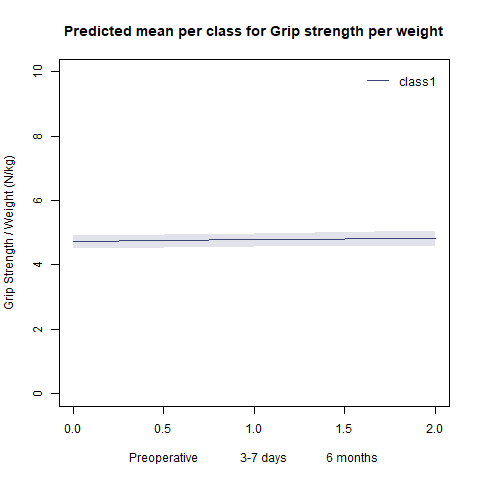

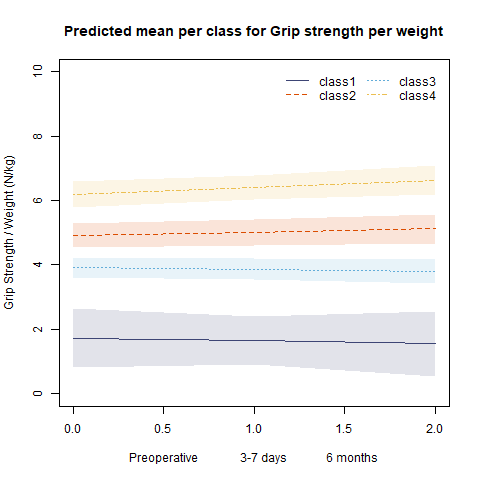

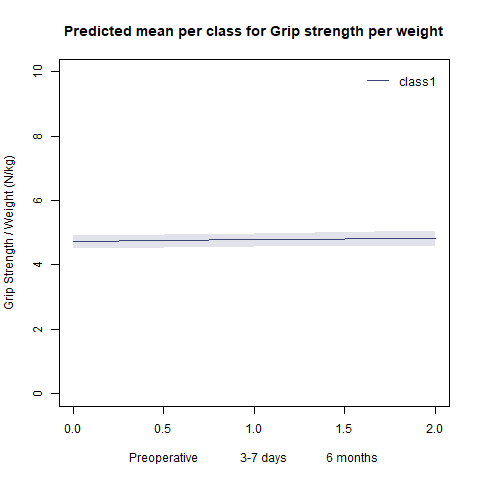

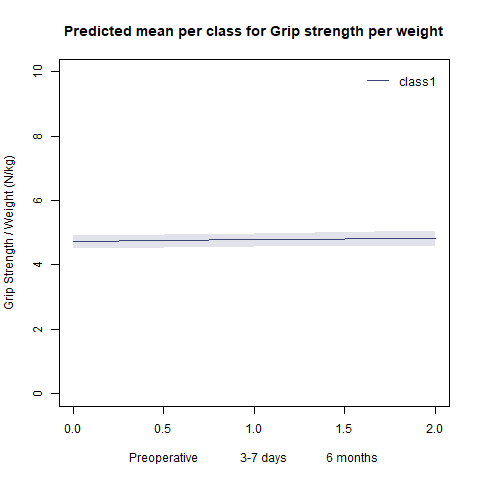

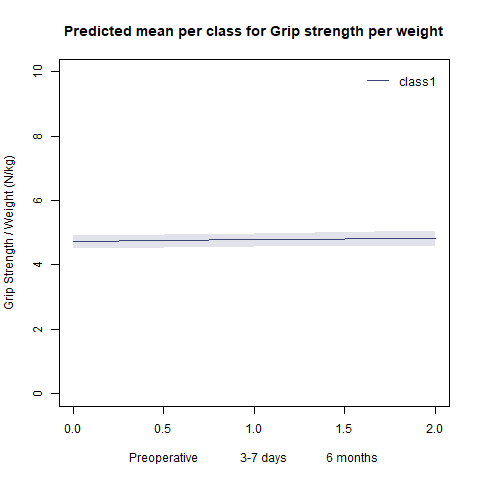

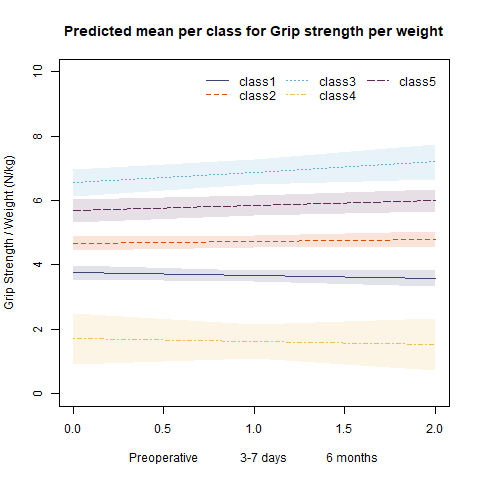

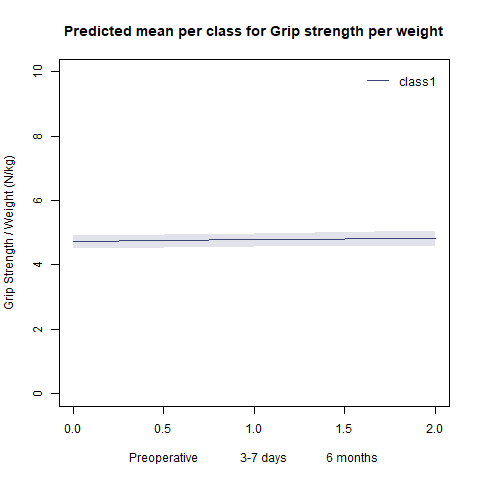

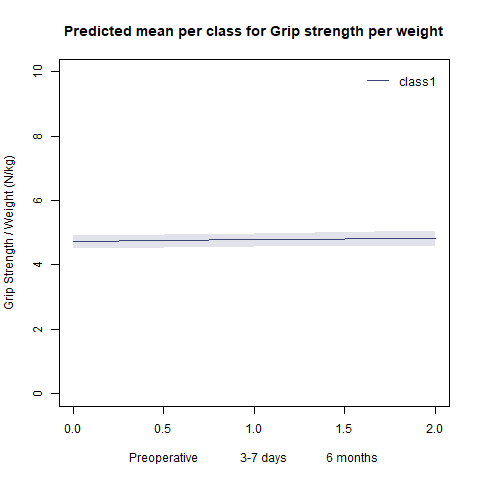

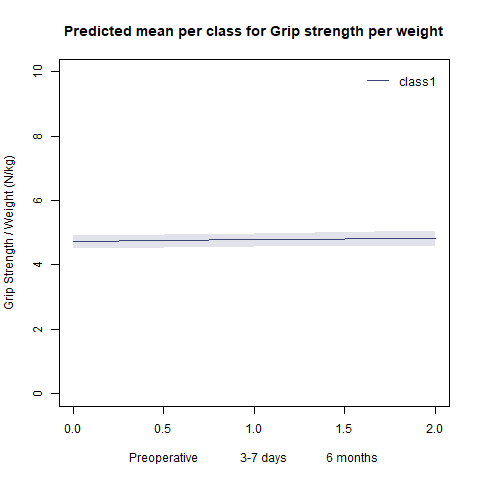


Figure S1: predicted mean with 95% confidence interval of linear grip strength per weight trajectories with allowance of within-class variances in male, n=116


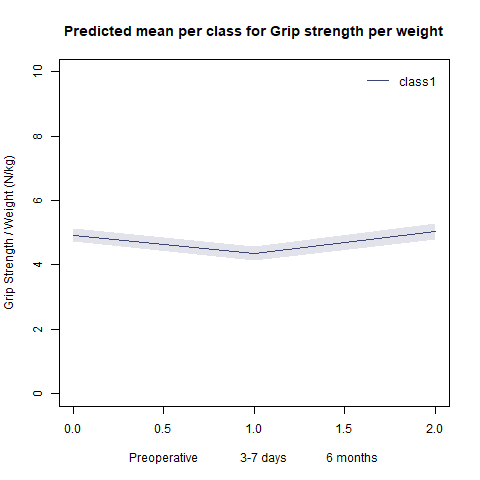

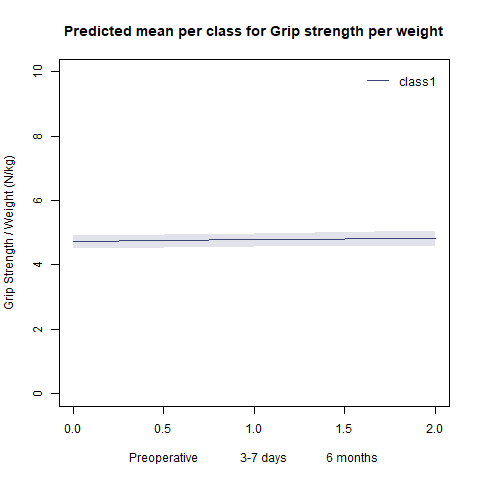

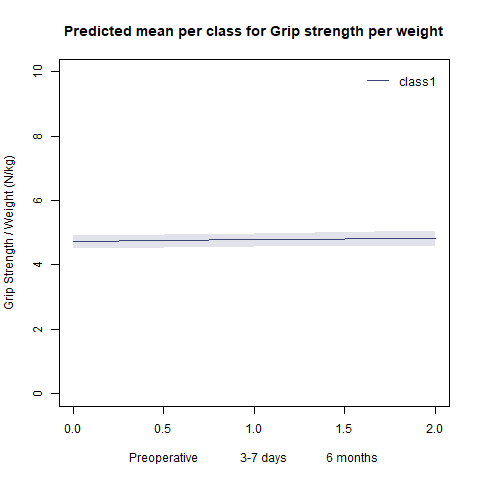

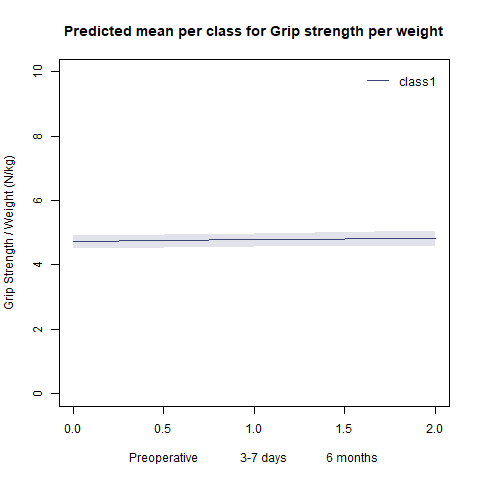

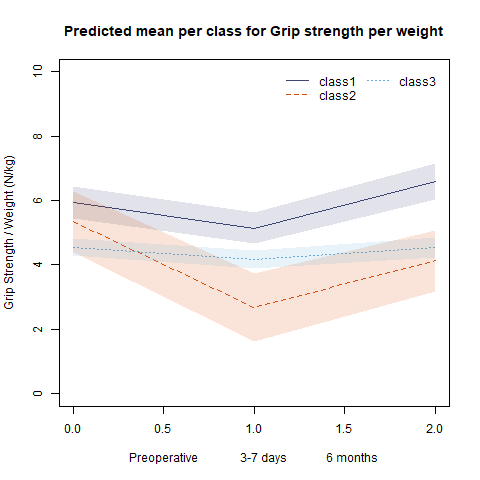

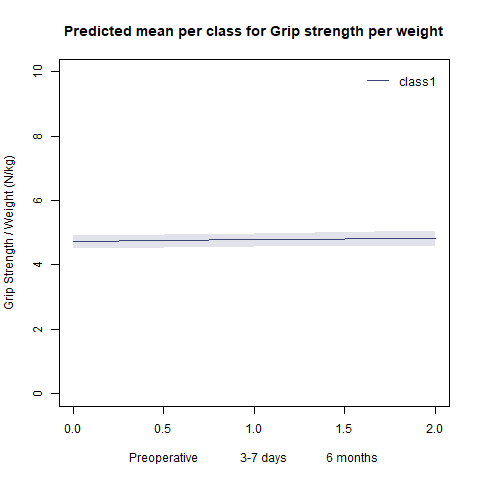

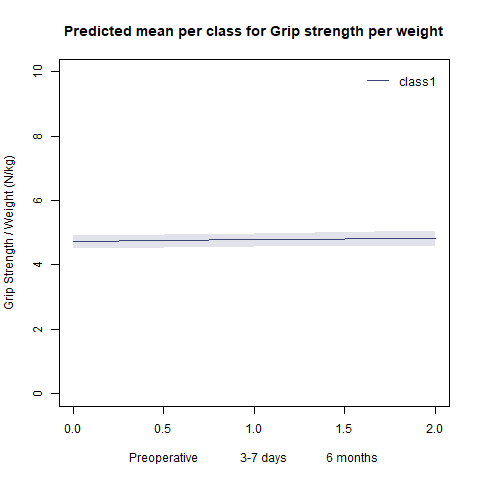

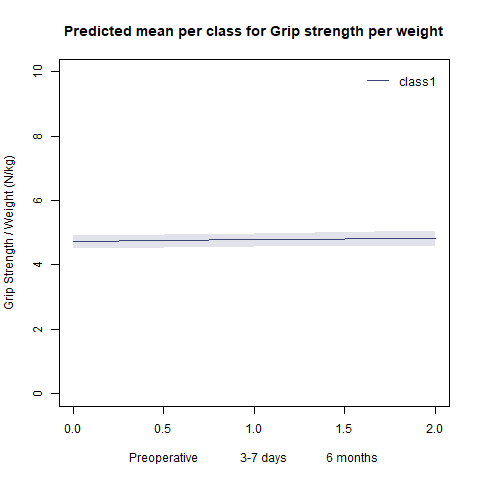

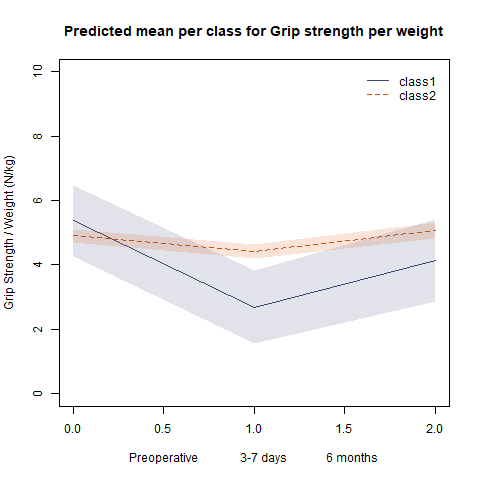

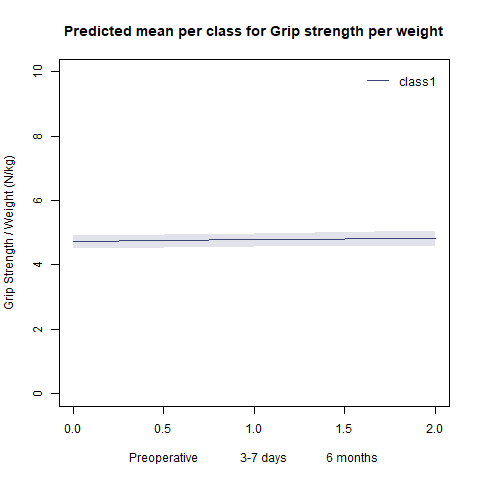

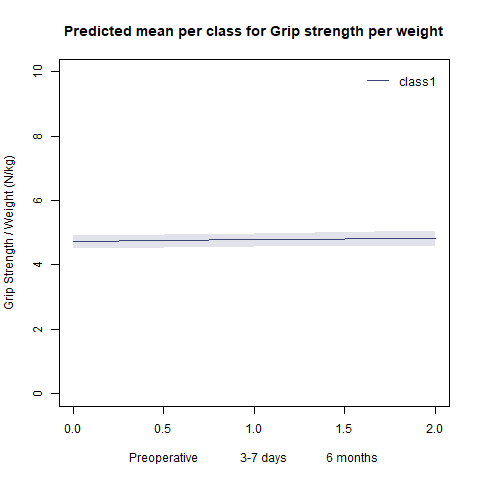

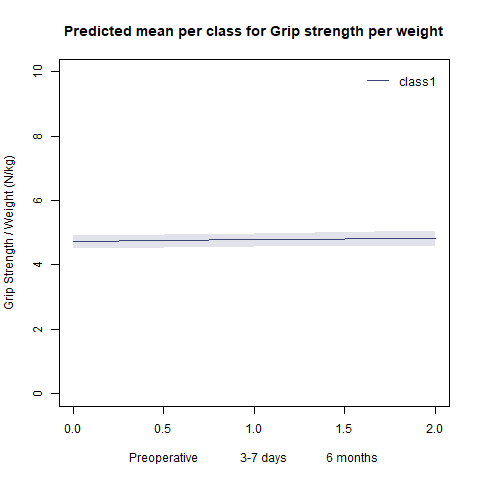

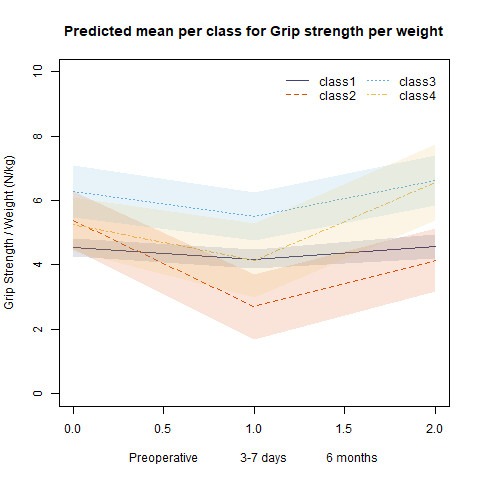

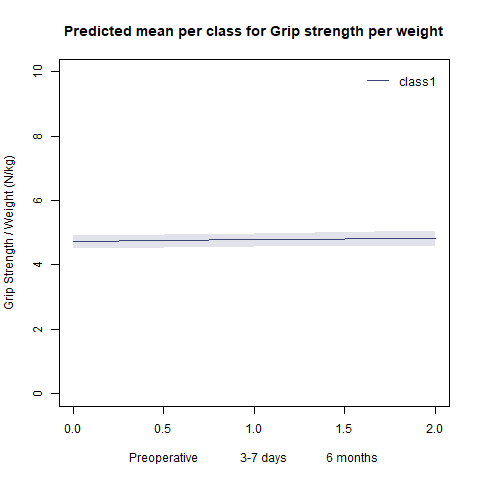

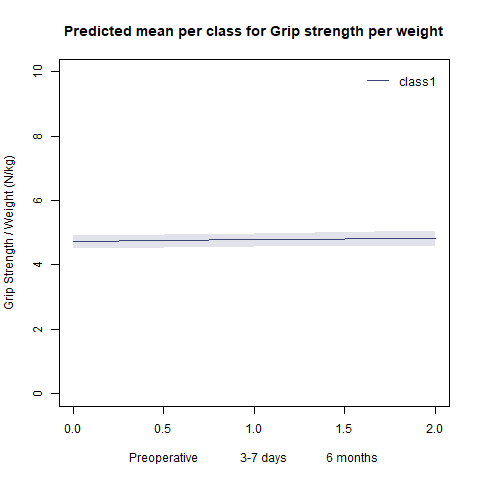

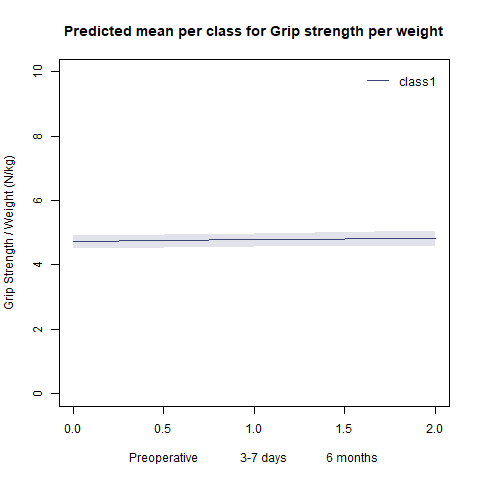

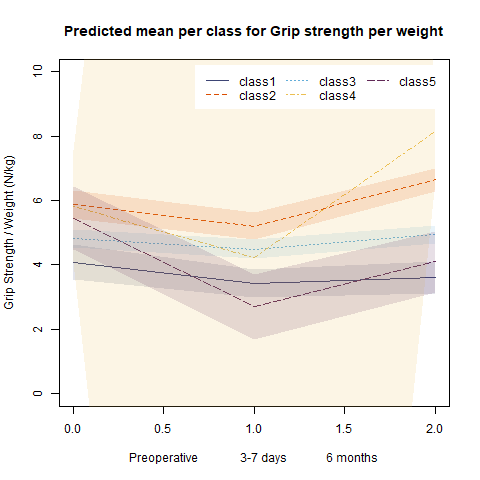

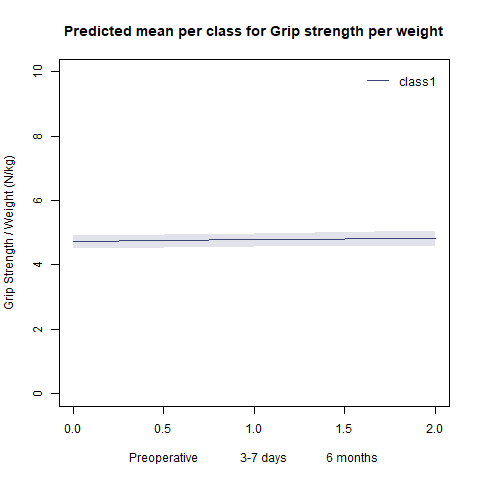

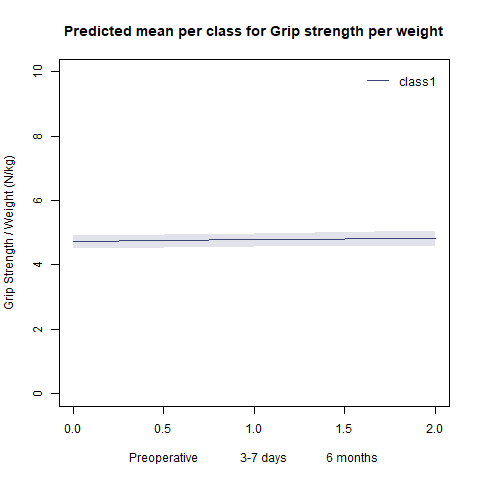

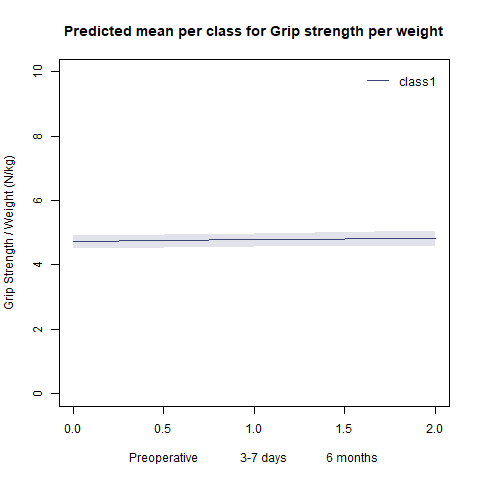


Figure S2: predicted mean with 95% confidence interval of quadratic grip strength per weight trajectories with allowance of within-class variances in male, n=116


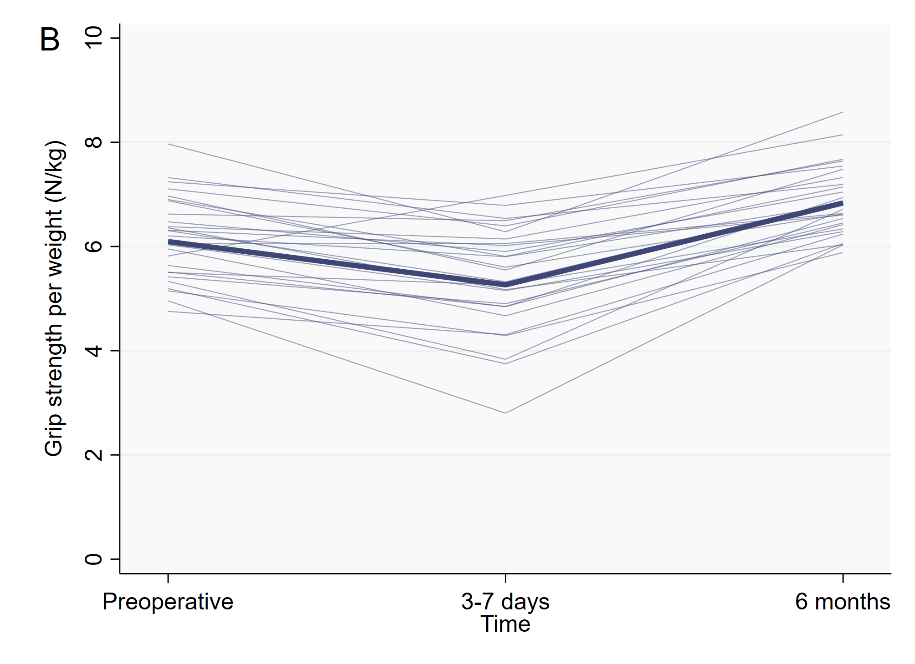

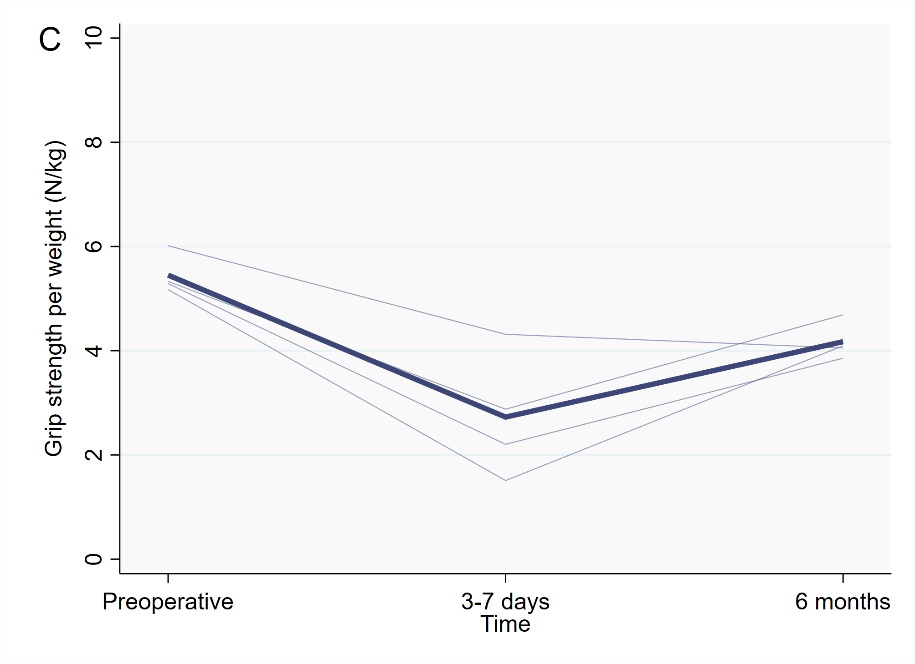

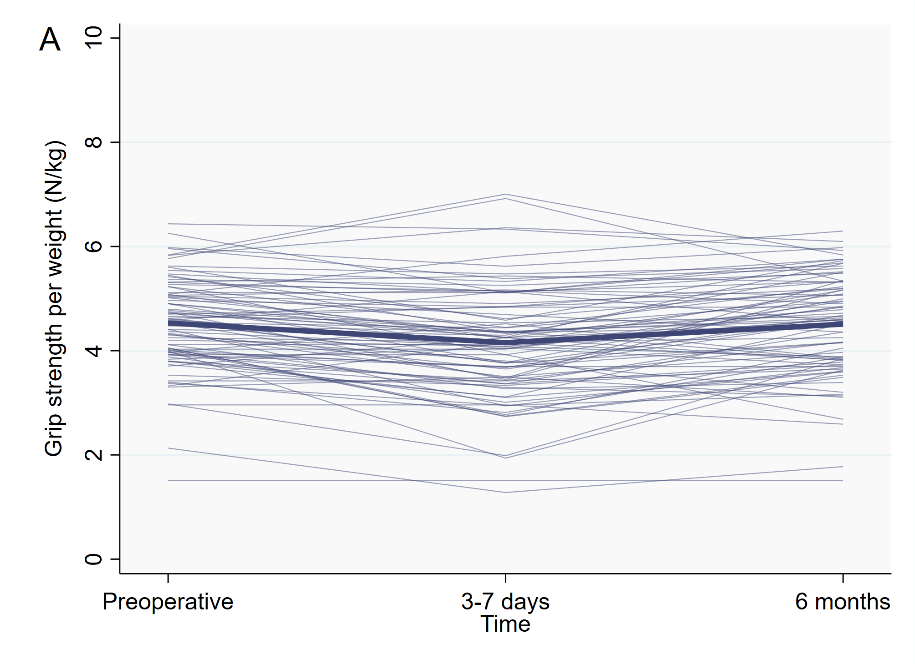


Figure S3: individual trajectories of grip strength per weight based on latent class growth mixture modelling in male. A) “Stable average” grip strength trajectory (n=85); B) “High” grip strength trajectory (n=27); C) “High-low” grip strength trajectory (n=4).


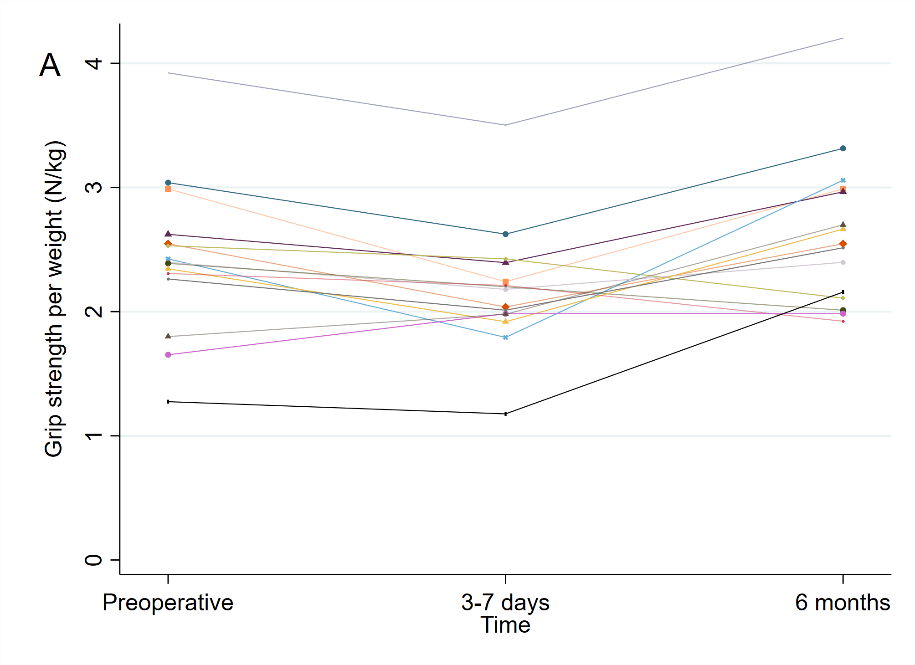

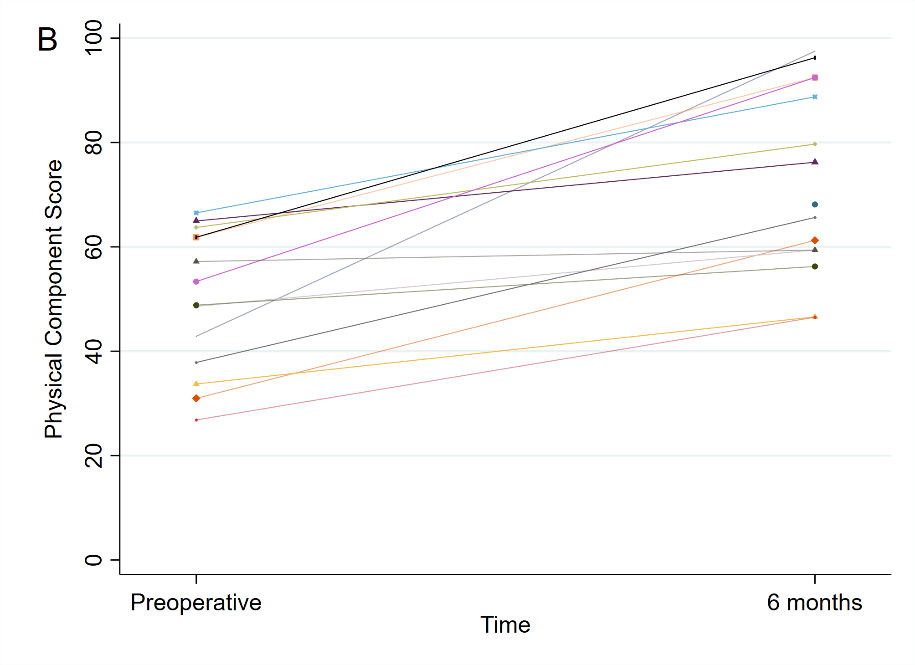

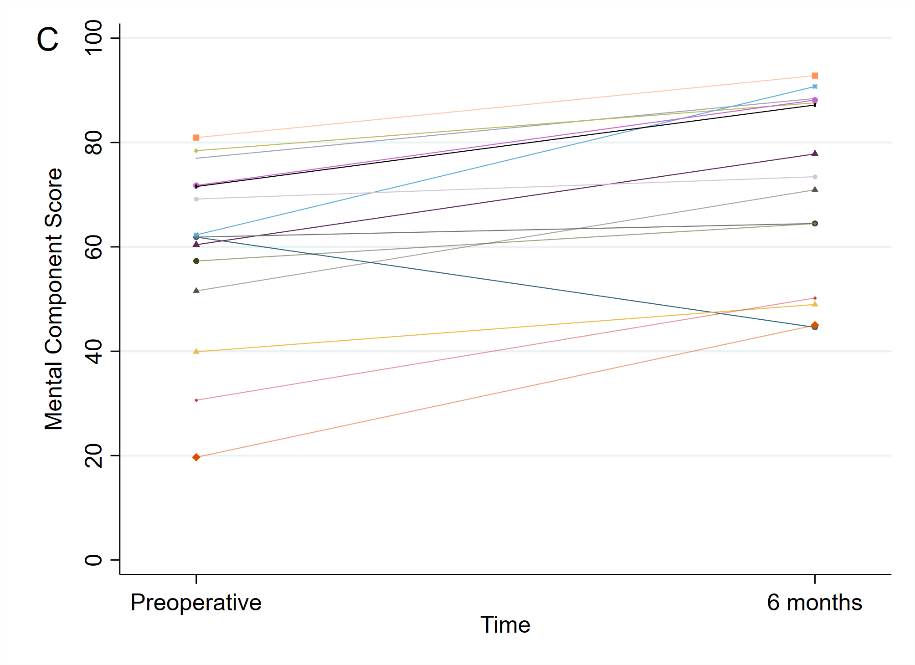


Figure S4: individual trajectories before and after coronary artery bypass grafting in female (n=15). A) grip strength per weight; b) Health-related Quality of Life, Physical component score; C) Health-related Quality of Life, Mental component score.
